# Re-exploring the association between the central venous pressure and the risk of sepsis-associated acute kidney injury according to the latest definition: Analysis of the MIMIC-IV database

**DOI:** 10.12669/pjms.41.5.12047

**Published:** 2025-05

**Authors:** Yingxiu Liu, Baiqing Ren, Muqiao Cheng, Junming Du, Rongrong Ren

**Affiliations:** 1 Yingxiu Liu, M.D Department of Anesthesiology and Surgical Intensive Care Unit, XinHua Hospital Affiliated with Shanghai Jiao Tong University School of Medicine, Shanghai, People’s Republic of China; 2 Baiqing Ren, M.D Department of Anesthesiology and Surgical Intensive Care Unit, XinHua Hospital Affiliated with Shanghai Jiao Tong University School of Medicine, Shanghai, People’s Republic of China; 3 Muqiao Cheng, M.D Department of Anesthesiology and Surgical Intensive Care Unit, XinHua Hospital Affiliated with Shanghai Jiao Tong University School of Medicine, Shanghai, People’s Republic of China; 4 Juanming Du Department of Anesthesiology and Surgical Intensive Care Unit, XinHua Hospital Affiliated with Shanghai Jiao Tong University School of Medicine, Shanghai, People’s Republic of China; 5 Rongrong Ren Department of Anesthesiology and Surgical Intensive Care Unit, XinHua Hospital Affiliated with Shanghai Jiao Tong University School of Medicine, Shanghai, People’s Republic of China

**Keywords:** Central Venous Pressure, Mortality, Sepsis-Associated Acute Kidney Injury

## Abstract

**Objective:**

The lack of a standard definition for sepsis-associated acute kidney injury (SA-AKI) makes the association between central venous pressure (CVP) and SA-AKI risk unclear. This study analyzed the relationship between CVP levels and the incidence and mortality of SA-AKI based on the most recent definition of the disease.

**Methods:**

This retrospective observational study utilized clinical records of sepsis patients from 2008 to 2019 admitted to the critical care unit (ICU) and in the Medical Information Mart for Intensive Care IV (MIMIC-IV) database were included. Based on the Acute Disease Quality Initiative (ADQI) definition of SA-AKI, patients were stratified into SA-AKI and non-SA-AKI groups. Patients were further categorized into four groups based on the CVP levels by the optimal prediction of SA-AKI incidence retrospectively. Cox proportional hazards models and a restricted cubic splines (RCS) model were employed to evaluate the relationship between CVP levels and SA-AKI risk. Additionally, Kaplan-Meier survival analysis was conducted to compare disparities in primary and secondary endpoints across groups stratified by CVP levels.

**Results:**

A total of 6129 patients were included. An independent relationship was observed between CVP levels and the risk of SA-AKI (p <0.001). Cox proportional hazards analysis demonstrated that SA-AKI incidence increased by 33% in patients with CVP≥10.19mmHg and 48% in patients with CVP≥13.67mmHg compared to patients with CVP<6.87mmHg. RCS analysis demonstrated a U-shaped association between CVP levels and mortality. In addition, the 90-day mortality risk decreased when CVP was between 4.89 and 13.12 mmHg (p< 0.001).

**Conclusion:**

Elevated CVP levels are associated with the occurrence of SA-AKI in sepsis patients. Maintaining CVP levels between 4.89mmHg and 10.19mmHg may help reduce the incidence and mortality of SA-AKI.

## INTRODUCTION

Sepsis is associated with high morbidity and mortality and often leads to severe organ dysfunction, including acute kidney injury (AKI).^1^ Indeed, studies show that sepsis is responsible for 25-75% of all AKI incidences.^2^,^3^ However, there is still no standardized definition of sepsis-associated acute kidney injury (SA-AKI). In 2023, the Acute Disease Quality Initiative (ADQI) workgroup released a consensus definition of SA-AKI^4^ that combines the diagnosis of sepsis, defined by the Sepsis 3.0 criteria,^1^ with the presence of AKI, as determined by the Kidney Disease Improving Global Outcomes (KDIGO) criteria,^5^ occurring within seven days of the sepsis diagnosis.

Fluid and resuscitation therapy is a key treatment strategy for sepsis that aims to increase preload and cardiac output to maintain adequate oxygen delivery to vital organs.^6^ Central venous pressure (CVP) is generally used for assessing volume status and responsiveness at the bedside. Several studies have reported that elevated CVP may increase renal venous pressure and decrease renal perfusion and glomerular filtration rate in critically ill patients and experimental models, leading to AKI.^7^-^9^ However, the association between CVP and SA-AKI is still unclear due to the lack of a standard definition for SA-AKI.

This study aimed to assess the association of CVP and SA-AKI in the critical care unit (ICU) using the publicly available clinical database Medical Information Mart for Intensive Care IV (MIMIC-IV)^10^ and based on the ADQI 28 Workgroup’s consensus definition of SA-AKI. We hypothesize that elevated CVP is associated with SA-AKI development and worse outcomes.

## METHODS

This retrospective observational study utilized data from the publicly available MIMIC-IV database, specifically the records of ICU patients between the years 2008 to 2019.

### Ethics approval and consent:

**Supplementary Fig.1:**
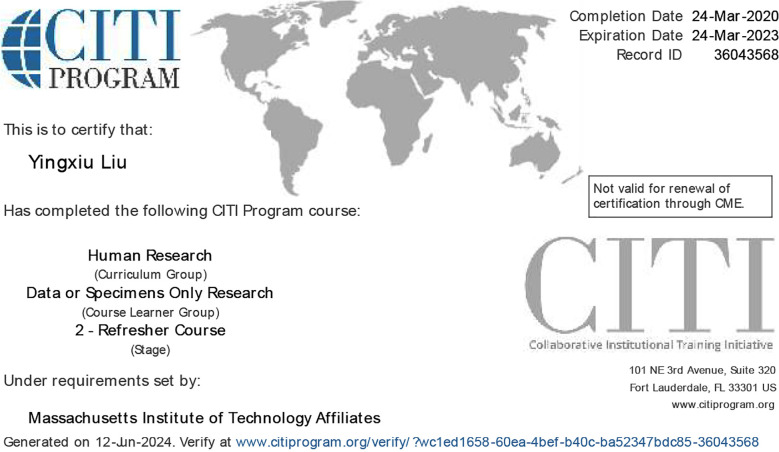
CITI completion certificate.

To comply with relevant regulations, the author (Record ID 36043568) obtained both a Collaborative Institutional Training Initiative (CITI) certification and the necessary permissions to utilize the MIMIC-IV database (Supplementary Fig.1). MIMIC-IV database used in the present study was approved by the Institutional Review Boards (IRB) of Institutional Review Boards of Beth Israel Deaconess Medical Center and the Massachusetts Institute of Technology and was given a waiver of ethics approval statement and informed consent.

### Inclusion criteria:

The study population in MIMIC-IV comprised 7,799 adult (≥18 years of age) patients diagnosed with sepsis (based on the diagnostic guidelines of sepsis-3) and with recorded CVP levels, admitted to the ICU. In cases where patients had multiple admissions, only the initial stay was considered. SA-AKI was diagnosed based on the 28^th^ ADQI workgroup consensus definition of SA-AKI.

### Exclusion criteria:

- Pre-existing chronic kidney disease (CKD).
- AKI diagnosis without detailed timing records.
- AKI occurring before the sepsis diagnosis.
- Diagnosed with AKI more than seven days after sepsis diagnosis.
- Erroneous survival times.

**Supplementary Fig.2:**
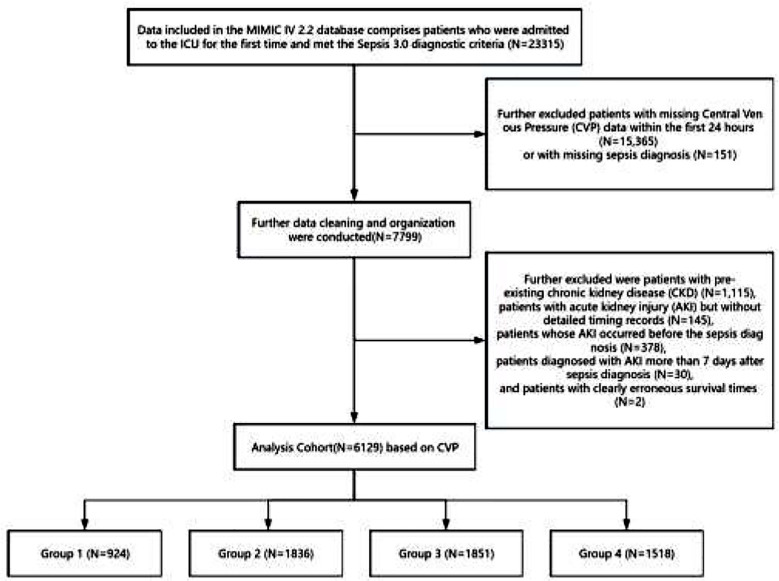
Flow diagram of patients included in the trial.

Ultimately, a final study cohort of 6,129 patients was established and divided into four groups (Supplementary Fig.2). The grouping was determined by the optimal prediction of SA-AKI incidence using univariate analysis of CVP as a four-level factor, with the area under the curve (AUC) serving as the statistical criterion. The ranges for the four groups were as follows: Group-1: 0-6.87mmHg; Group-2: 6.87-10.19mmHg; Group-3: 10.19-13.67mmHg; Group-4: 13.67-23.31mmHg. Patients were further divided into two groups based on the incidence of SA-AKI.

### Data collection and analyses:

The primary endpoint of interest was the incidence of SA-AKI. The hemodynamic parameters of sepsis patients were recorded in the first 24 hours after admission. Cox proportional hazard models were used to calculate the hazard ratios (HRs) and 95% confidence intervals (CIs) for the association between CVP levels and the incidence of AKI, adjusting for multiple variables. Secondary endpoints included overall mortality, 28-day mortality, and 90-day mortality rates for the entire study population and the subset of patients with SA-AKI. Based on CVP levels, Kaplan-Meier survival analysis and log-rank tests were employed to estimate the incidence of AKI and overall mortality, as well as 28-day and 90-day mortality across different CVP groups. HRs were calculated and results were presented with 95% CIs. All four models used the lowest CVP level group as the reference group.

### Statistical analysis:

Continuous variables were described using mean (standard deviation [SD]) or median (interquartile range [IQR]). Group comparisons were performed using the Mann-Whitney U test or Student’s *t*-test. Categorical variables were presented as frequencies and percentages (%), and group comparisons were conducted using Fisher’s exact test or the chi-square test. Additionally, restricted cubic spline models were employed to explore potential dose-response relationships between CVP and the incidence of AKI, as well as 28 days and 90 days mortality, adjusting for the variables mentioned above. All data analyses were performed using R version 4.4.0. A two-sided p-value of less than 0.05 was considered statistically significant.

## RESULTS

### Baseline characteristics:

This retrospective study included records of 6,129 patients with a median age of 67. 64% were male; the median CVP was 10.7mmHg, and the incidence of SA-AKI in the cohort was 77.5%. The baseline characteristics of patients stratified by the occurrence of SA-AKI. SA-AKI was associated with a higher rate of mechanical ventilation, longer hospital and ICU stays, and higher mortality rates (*p*<0.001) are presented in Table-I.

**Table-I:** Baseline characteristics stratified by SA-AKI occurrence.

Variable	SA-AKI			p-value^2^
	Overall, N = 6,129^1^	No, N = 1,375^1^	Yes, N = 4,754^1^	
Age				<0.001
Median [IQR]	67 [58, 76]	65 [56, 74]	68 [58, 77]	
Gender				0.011
Male	3,904 (64%)	916 (67%)	2,988 (63%)	
Female	2,225 (36%)	459 (33%)	1,766 (37%)	
BMI				<0.001
Median [IQR]	28.6 [25.3, 32.6]	27.0 [24.0, 30.0]	29.2 [25.7, 33.3]	
WBC				<0.001
Median [IQR]	15.1 [11.6, 19.3]	14.3 [11.0, 18.2]	15.3 [11.7, 19.6]	
Platelet				0.009
Median [IQR]	180 [141, 232]	176 [141, 220]	181 [141, 235]	
Hemoglobin				>0.9
Median [IQR]	11.30 [10.30, 12.50]	11.40 [10.30, 12.40]	11.30 [10.30, 12.50]	
Lactate				<0.001
Median [IQR]	2.70 [1.90, 3.90]	2.50 [1.85, 3.30]	2.80 [2.00, 4.20]	
Ureanitrogen				<0.001
Median [IQR]	19 [14, 29]	16 [13, 21]	20 [15, 31]	
Creatinine				<0.001
Median [IQR]	1.00 [0.80, 1.40]	0.90 [0.70, 1.10]	1.00 [0.80, 1.50]	
SOFA				<0.001
Median [IQR]	6 [4, 9]	5 [3, 7]	6 [4, 9]	
APS III				<0.001
Median [IQR]	40 [29, 62]	33 [25, 47]	44 [31, 66]	
HR				0.2
Median [IQR]	102 [90, 116]	101 [91, 114]	102 [90, 117]	
MAP				<0.001
Median [IQR]	59 [54, 63]	60 [55, 64]	59 [54, 63]	
RR				<0.001
Median [IQR]	27.0 [24.0, 31.0]	26.0 [23.0, 30.0]	27.0 [24.0, 32.0]	
CVP				<0.001
Median [IQR]	10.7 [8.1, 13.7]	9.3 [7.1, 11.9]	11.0 [8.5, 14.0]	
Hypertension	3,611 (59%)	785 (57%)	2,826 (59%)	0.12
DM	1,647 (27%)	303 (22%)	1,344 (28%)	<0.001
Heart Failure	1,452 (24%)	222 (16%)	1,230 (26%)	<0.001
Myocardial Infarction	357 (5.8%)	37 (2.7%)	320 (6.7%)	<0.001
Pneumonia	1,498 (24%)	272 (20%)	1,226 (26%)	<0.001
COPD	205 (3.3%)	22 (1.6%)	183 (3.8%)	<0.001
Heart Disease	613 (10%)	98 (7.1%)	515 (11%)	<0.001
Respiration				<0.001
0	2,988 (49%)	668 (49%)	2,320 (49%)	
1	885 (14%)	228 (17%)	657 (14%)	
2	1,396 (23%)	319 (23%)	1,077 (23%)	
3	679 (11%)	139 (10%)	540 (11%)	
4	181 (3.0%)	21 (1.5%)	160 (3.4%)	
Coagulation				<0.001
0	3,382 (55%)	714 (52%)	2,668 (56%)	
1	1,920 (31%)	495 (36%)	1,425 (30%)	
2	695 (11%)	145 (11%)	550 (12%)	
3	109 (1.8%)	19 (1.4%)	90 (1.9%)	
4	23 (0.4%)	2 (0.1%)	21 (0.4%)	
Liver				<0.001
0	5,444 (89%)	1,276 (93%)	4,168 (88%)	
1	247 (4.0%)	51 (3.7%)	196 (4.1%)	
2	300 (4.9%)	42 (3.1%)	258 (5.4%)	
3	73 (1.2%)	4 (0.3%)	69 (1.5%)	
4	65 (1.1%)	2 (0.1%)	63 (1.3%)	
Cardiovascular				<0.001
0	1,695 (28%)	414 (30%)	1,281 (27%)	
1	2,748 (45%)	634 (46%)	2,114 (44%)	
2	36 (0.6%)	3 (0.2%)	33 (0.7%)	
3	925 (15%)	201 (15%)	724 (15%)	
4	725 (12%)	123 (8.9%)	602 (13%)	
Neurologic				0.3
0	5,340 (87%)	1,180 (86%)	4,160 (88%)	
1	318 (5.2%)	73 (5.3%)	245 (5.2%)	
2	94 (1.5%)	24 (1.7%)	70 (1.5%)	
3	82 (1.3%)	17 (1.2%)	65 (1.4%)	
4	295 (4.8%)	81 (5.9%)	214 (4.5%)	
AKI stage				<0.001
0	1,375 (22%)	1,375 (100%)	0 (0%)	
1	1,374 (22%)	0 (0%)	1,374 (29%)	
2	2,337 (38%)	0 (0%)	2,337 (49%)	
3	1,043 (17%)	0 (0%)	1,043 (22%)	
AKI time after sepsis				<0.001
Median [IQR]	0.91 [0.50, 2.34]	7.00 [7.00, 7.00]	0.70 [0.43, 1.20]	
Input Fluid				<0.001
Median [IQR]	3,422 [950, 6,319]	2,603 [804, 4,514]	3,759 [1,007, 7,265]	
Ventilation	4,658 (76%)	942 (69%)	3,716 (78%)	<0.001
Ventilation day				<0.001
Median [IQR]	1 [1, 1]	1 [0, 1]	1 [1, 1]	
CRRT	319 (5.2%)	0 (0%)	319 (6.7%)	<0.001
Hospital Los Day				<0.001
Median [IQR]	7 [5, 12]	6 [4, 9]	8 [5, 14]	
ICU Los Day				<0.001
Median [IQR]	2.3 [1.3, 4.7]	1.3 [1.1, 2.2]	2.9 [1.5, 5.7]	
Mortality of 28 day	750 (12%)	70 (5.1%)	680 (14%)	<0.001
Mortality of 90 day	952 (16%)	96 (7.0%)	856 (18%)	<0.001
In-hospital Dead	696 (11%)	63 (4.6%)	633 (13%)	<0.001
Dead	1,626 (27%)	218 (16%)	1,408 (30%)	<0.001
Norepinephrine				<0.001
Median [IQR]	0 [0, 4]	0 [0, 0]	0 [0, 7]	
Phenylephrine				<0.001
Median [IQR]	2 [0, 32]	1 [0, 12]	2 [0, 42]	
CVP group				<0.001
Q1	924 (15%)	328 (24%)	596 (13%)	
Q2	1,836 (30%)	495 (36%)	1,341 (28%)	
Q3	1,851 (30%)	361 (26%)	1,490 (31%)	
Q4	1,518 (25%)	191 (14%)	1,327 (28%)	

1 n (%)

2 Wilcoxon rank sum test; Pearson’s Chi-squared test

SA-AKI: sepsis-associated acute kidney injury; BMI: body mass index; WBC: white blood cell count; DM: diabetes mellitus; COPD: chronic obstructive pulmonary disease; HR: heart rate; MAP: mean arterial pressure; RR: respiratory rate; SOFA: Sequential Organ Failure Assessment; CRRT: continuous renal replacement therapy.

### Primary endpoint:

**Fig.1:**
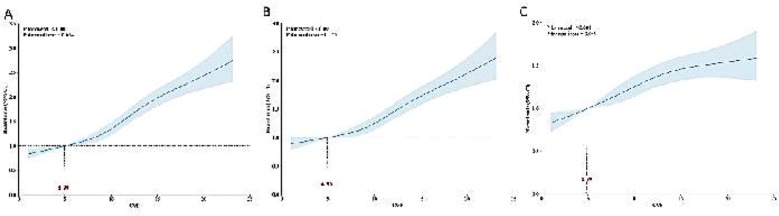
Restricted cubic spline curves for the occurrence of SA-AKI hazard ratio. (A): Model-1 (B): Model-2 (C): Model-3.

The restricted cubic spline regression model (Fig.1) demonstrates the dose-response relationship between CVP and the risk of SA-AKI in both unadjusted and fully adjusted models (overall p-value <0.001).

**Fig.2:**
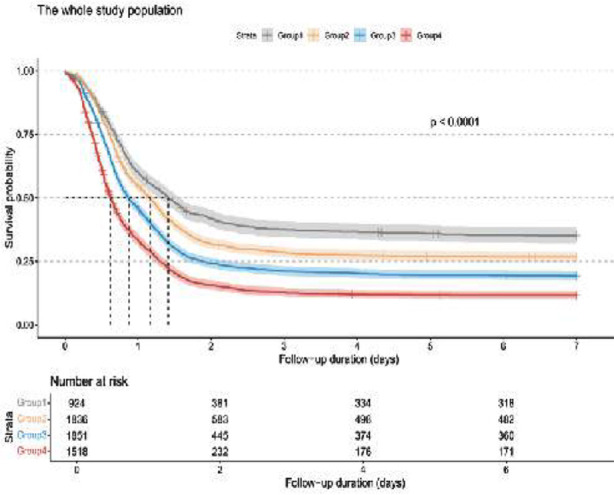
The Kaplan–Meier curves depict the occurrence of SA-AKI in the overall population.

The incidence of AKI differed significantly among the groups during the follow-up period is shown in Fig-2. Cox proportional hazards analysis showed a statistically significant association between CVP and the risk of AKI in all of unadjusted (Model-1), partially adjusted for sex, age, and BMI (Model-2), and the fully adjusted for WBC, platelet, hemoglobin, lactate, urea nitrogen, creatinine, hypertension, DM, heart failure, myocardial infarction, pneumonia, COPD, tuberculosis, heart disease, respiration, coagulation, liver, cardiovascular, neurologic, ventilation, ventilation day, CRRT, input fluid, HR, MAP, RR, norepinephrine, phenylephrine, SOFA, APS III (Model-3) models (*p*<0.001). Furthermore, when CVP was categorized into four groups, higher CVP groups were significantly associated with an increased risk of AKI in all of three models(*p*<0.001) (Table-II).

**Table-II:** Cox proportional hazard ratios (HR) for AKI incidence based on CVP.

Categories	Model 1		Model 2		Model 3	
	HR (95% CI)	P-value	HR (95% CI)	P-value	HR (95% CI)	P-value
AKI incidence						
Continuous variables per 1 unit	1.07(1.06, 1.07)	<0.001*	1.06(1.05, 1.07)	<0.001*	1.03(1.02, 1.04)	<0.001*
Group ^a^						
Group 1(N =924)	Ref.		Ref.		Ref.	
Group 2(N =1836)	1.25(1.13, 1.37)	<0.001*	1.18(1.07, 1.30)	<0.001*	1.15(1.04, 1.27)	0.006*
Group 3(N =1851)	1.59(1.45, 1.75)	<0.001*	1.42(1.29, 1.57)	<0.001*	1.33(1.20, 1.46)	<0.001*
Group 4(N =1518)	2.23(2.02, 2.46)	<0.001*	1.94(1.76, 2.14)	<0.001*	1.48(1.33, 1.64)	<0.001*

Model-1 was unadjusted. Model 2 was adjusted for sex, age, and BMI. Model 3 was adjusted for the variables in model 2 and further adjusted for WBC, platelet, hemoglobin, lactate, urea nitrogen, creatinine, hypertension, DM, heart failure, myocardial infarction, pneumonia, COPD, tuberculosis, heart disease, respiration, coagulation, liver, cardiovascular, neurologic, ventilation, ventilation day, CRRT, input fluid, HR, MAP, RR, norepinephrine, phenylephrine, SOFA, APS III.

a CVP (cm H_2_O), Group 1: 0-6.87; Group 2: 6.87-10.19; Group 3: 10.19-13.67; Group 4: 13.67-23.31.

### Secondary endpoints:

**Fig.3:**
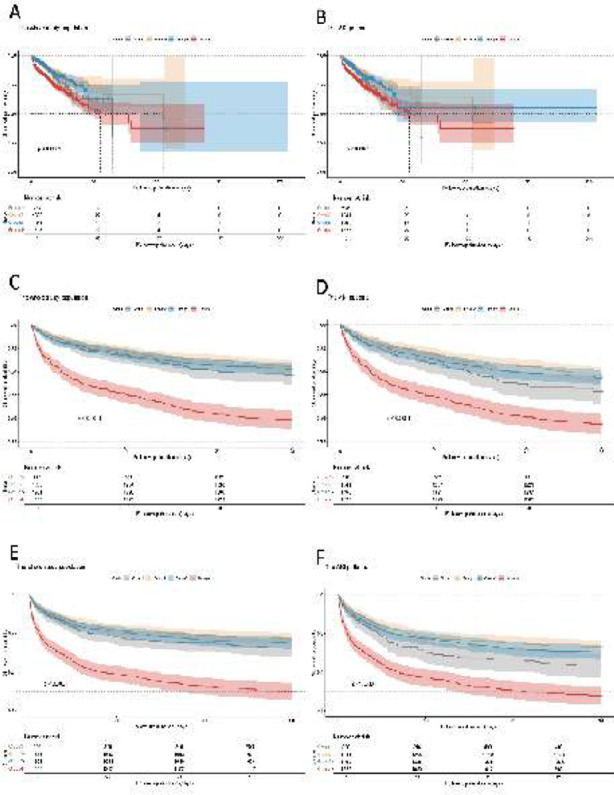
Restricted cubic spline curves for the 28-day mortality (A,B&C) and 90-day mortality (D,E&F) hazard ratio. (A&D): Model-1(B&E): Model-2 (C&F): Model-3.

**Fig.4:**
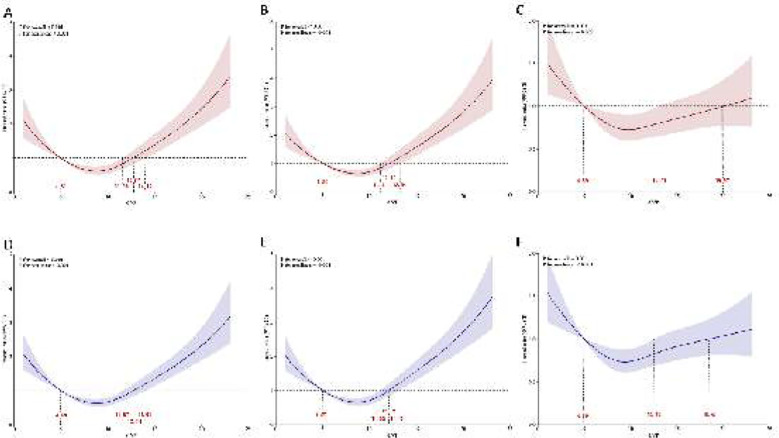
Kaplan–Meier survival analysis curves illustrate total, 28-day and 90-day mortality in the entire population (A,C&E) and in the SA-AKI population (B,D&F).

As shown in Fig.3, RCS analysis results revealed a U-shaped association between CVP levels and a 28-day (Fig.3A- C) and 90-day (Fig.3 D-F) mortality (*p* for non-linearity < 0.001). A similar trend was detected for the non-adjusted, partially adjusted, and fully adjusted models: the risk of death decreased with the rising CVP when CVP<4.89 mmHg. When CVP was > 4.89 mmHg, the risk initially decreased and then increased with the rising CVP. After fully adjusted, the results indicated that the 28-day mortality risk decreased when CVP was between 4.89 and 14.01 mmHg (Fig.3C), and the 90-day mortality risk decreased when CVP was between 4.89 and 13.12 mmHg (Fig.3F). Group-4 patients had a significantly higher mortality rate compared to the other three groups (*p* < 0.05) (Fig.4A, C, E). No significant differences were found in the other three groups (*p* > 0.05). Similar trends were observed in patients with SA-AKI (*p*<0.05) (Fig.4B, D, F).

## DISCUSSION

To the best of our knowledge, this is the first study investigating the association of CVP and SA-AKI in sepsis patients according to the new consensus ADQI definition. The results of this study indicated a significant association between higher CVP and the development of SA-AKI in critically ill patients with sepsis. Furthermore, elevated CVP significantly correlated with in-hospital, 28-day, and 90-day mortality both in the entire cohort and the subgroup of SA-AKI patients.

According to a recent multicenter study involving 90000 patients, about 16.7% of septic patients will develop SA-AKI in the ICU, often within the first day of sepsis onset,^11^ which is consistent with the result of this study. Multiple mechanisms contribute to SA-AKI development, including Cytokine Release Syndrome (CRS), systemic and renal inflammation, Hyper-inflammatory Syndrome, Renin-Angiotensin-aldosterone System (RAAS), mitochondrial dysfunctions, metabolic reprogramming, and microcirculatory abnormalities.^4^,^12^-^15^ In addition, multiple studies have shown that fluid overload following resuscitation may affect renal microcirculation, increases the incidence and mortality of AKI in critically ill patients.^16^,^17^

The research shows that elevated CVP and renal venous pressure can activate the sympathetic nervous system and the RAAS, promoting the reabsorption of water and sodium by the renal tubules and further exacerbating renal congestion.^18^ At the same time, elevated CVP and renal venous pressure further reduce renal blood flow, potentially contributing to SA-AKI.^19^,^20^

Due to the absence of a standardized definition of SA-AKI, the inclusion criteria for SA-AKI in different articles are inconsistent.^21^,^22^ Therefore, the optimal CVP level remains clinically debated. A study by Legrand et al.^23^ showed that in septic patients, the best hemodynamic goals were achieved when the CVP was less than 8-12 mmHg, and the incidence of new or persistent AKI sharply increased with the increase of CVP over this limit. Other studies showed that in patients after cardiac surgery, CVP exceeding 10-10.9 mmHg is associated with a 0.5-5 times increase in the risk of AKI for every additional One mmHg.^24^-^26^

This study explored the association between CVP and the risk of SA-AKI according to the latest definition. We concluded that maintaining CVP between 4.89mmHg and 11.56mmHg was beneficial to reduce the mortality of patients. In addition, CVP≥10.19mmHg and CVP ≥13.67mmHg were associated with a 33% and 48% increase in the incidence of SA-AKI, respectively, compared to septic patients with CVP below 6.87mmHg. These results suggest that a lower CVP level is beneficial to avoid SA-AKI. Therefore, the existing guidelines may be updated to recommend maintaining target CVP at 4.89mmHg-10.19mmHg to reduce the occurrence of SA-AKI and lower the mortality rate of septic patients during fluid resuscitation. Nevertheless, further prospective clinical trials are needed to confirm our results.

### Limitations:

This study has several limitations. Since this is a retrospective study of data retrieved from the public database, the cohort could not represent all sepsis patients. Additionally, there is a risk of incomplete data and outliers. We employed multiple imputations via weighted predictive mean matching to maintain statistical robustness, thereby mitigating the potential bias associated with missing data.

## CONCLUSIONS

In septic patients, the occurrence of SA-AKI and mortality rates increase with the rise of CVP. Maintaining CVP levels between 4.89 mmHg and 10.19 mmHg may reduce the incidence of developing SA-AKI and improve survival of septic patients.

### Authors’ Contributions:

**YL** and **BR**: Concept, study design, literature search and manuscript writing.

**MC** and **JD**: Data collection, data analysis interpretation. Critical review.

**RR:** Manuscript revision and validation. Critical analysis.

All authors have read and approved the final manuscript.
